# Tissue-Specific Fluorescent Protein Turnover in Free-Moving Flies

**DOI:** 10.3390/insects16060583

**Published:** 2025-05-31

**Authors:** Katherine S. Bell, Sebastian Ko, Sam Ali, Brett Bognar, Michael Khmelkov, Nick Rau, Oscar K. Peng, Mavi Eyuboglu, John Paine, Andy Tong, Anuj Saria, Siddharth Agrawal, Kelvin J. A. Davies, John Tower

**Affiliations:** 1 Molecular and Computational Biology Section, Department of Biological Sciences, University of Southern California, Los Angeles, CA 90089, USA; bellkath@usc.edu (K.S.B.); kosebast@usc.edu (S.K.); sammiali.usc@gmail.com (S.A.); bognar@usc.edu (B.B.); khmelkov@usc.edu (M.K.); nickrau24@gmail.com (N.R.); oscar.peng@ucsf.edu (O.K.P.); meyubogl@usc.edu (M.E.); johnpain@usc.edu (J.P.); andytong@usc.edu (A.T.); anujsari@usc.edu (A.S.); siddharth.agrawal18@gmail.com (S.A.); 2 Leonard Davis School of Gerontology, Ethel Percy Andrus Gerontology Center, University of Southern California, Los Angeles, CA 90089, USA; kelvin@usc.edu; 3 Department of Biochemistry & Molecular Medicine, Keck School of Medicine of USC, University of Southern California, Los Angeles, CA 90089, USA

**Keywords:** *Drosophila*, GFP, DsRED, video, turnover, in vivo, degradation, proteasome, bortezomib, cycloheximide

## Abstract

Conditional transgenic systems and multi-copy target transgenes were used to produce transient fluorescent protein expression in adult *Drosophila melanogaster*, with the goal of developing an in vivo assay of protein turnover. Free-moving flies were assayed at multiple time points using video, and decay in fluorescence was used to calculate protein half-life. Additional experiments involved image capture of anesthetized flies. The half-life of eGFP was increased by the proteasome inhibitor bortezomib, both in vivo and in vitro, indicating proteasomal degradation of eGFP. The accumulation of eGFP in vivo was decreased by the protein synthesis inhibitor cycloheximide, without affecting half-life. The half-lives of several fluorescent proteins were determined, using both tissue-general and tissue-specific expression, in flies of both sexes and varying ages. Typical half-life values varied by fluorescent protein. DsRED showed a greater half-life than eGFP, and little if any degradation was detected for mCherry. Half-life also varied by tissue, with greater eGFP half-life observed in muscle relative to other tissues. Increased half-life with age was detected for DsRED but not for eGFP. Limited effects were observed for sex and female mating status. Taken together, the data indicate the in vivo assays are promising tools for the study of protein degradation regulated by protein sequence, subcellular compartment, tissue and small molecules.

## Simple Summary

The regulated synthesis and degradation of proteins is called protein turnover, and protein turnover is critical for the removal of damaged proteins and for normal regulation of cell and animal physiology. Defects in protein turnover during aging are implicated in the accumulation of damaged proteins in various tissues and in aging-associated diseases. In the past, protein turnover has typically been assayed by labeling the cells or animal with a radioactive or heavy isotope, followed by quantification by mass spectrometry. One limitation of these assays is that they are invasive and destructive, requiring the sacrifice of the animal. Here, we report the use of fluorescent proteins to assay protein half-life in vivo in adult *Drosophila melanogaster*. The ability to measure turnover without sacrifice of the animal facilitates the study of the effects of drugs and other small molecules.

## 1. Introduction

In the past, protein half-life has typically been assayed by labeling with a radioactive or heavy isotope followed by quantification by mass spectrometry [1]. One limitation of these assays is that they are invasive and destructive, requiring sacrifice of the animal. Here, we report the use of fluorescent proteins to assay protein half-life in vivo in adult *Drosophila melanogaster*.

Flies were engineered to produce transient expression of a fluorescent protein using the conditional transgenic systems Tet-ON or Gene-Switch [2,3,4]. After removal of the activating drug, synthesis of new protein ceases, and fluorescence declines in a manner proportional to protein degradation, yielding a measurement of protein half-life [1,5]. The conditional transgenic systems also allow for tissue-specific expression of fluorescent proteins, which is important in light of evidence for tissue-specific effects of aging on protein degradation [6,7,8]. Moreover, the system allows for assay of proteins targeted to organelles.

## 2. Materials and Methods

### 2.1. Video Assays

For video assay, flies were gently knocked into 4 dram (~15 mL) glass vials, using 6 flies per vial and 4 vials per group, and each vial was covered with a glass coverslip. Each vial was individually placed in a dark box illuminated using two LED lights, and two synchronized video cameras were used to capture fluorescence in 4-min videos (see Supplementary Figure S1). Prior to recording, flies were transferred to media supplemented with 200 µg/mL mifepristone for the Gene-Switch system or 640 µg/mL doxycycline for the Tet-ON system to initiate fluorescent protein expression and then removed from the drug media after 24 or 48 h, as indicated. Recordings were taken immediately after removal from the drug (day 1) and every day over a period of 5–6 days. Videos were analyzed using FluoreScore version 1 software, which identifies each fly and quantifies pixel intensity in the area of the flies in each frame [9]. The data from the cameras was combined to yield a fluorescence intensity value for each vial at each time point, and average fluorescence intensity values for the 4 vials in each group were averaged. Fluorescence typically peaked at 24 h after cessation of drug treatment for the Gene-Switch system and at 4–5 days after cessation of drug treatment for the Tet-ON system, after which it decreased exponentially. The natural logarithm of each average fluorescence value was taken, the values for each group were plotted as ln(fluorescence) versus time, and the slope of the resulting linear regression from peak to minimum was used to calculate half-life.

### 2.2. Fly Culture and Strains

*Drosophila melanogaster* were cultured at 25 °C using a standard agar/dextrose/cornmeal/yeast media [10], and adult flies were passaged to fresh media every other day. *Actin-GS-255B* is previously described and contains the *Actin5C* gene promoter driving tissue-general expression of Gene-Switch [1]. The *repo-GS* strain drives Gene-Switch expression in glial cells and was generously provided by Amita Sehgal [11]. The *5966-GS* strain expresses Gene-Switch in midgut enterocytes and was generously provided by Pankaj Kapahi [12]. *Actin88F-GS* drives expression of Gene-Switch in flight muscle and was generously provided by Jason Karpac [13]. The *w[1118]*; *3xP3-GFP[M1]* strain transgenic construct includes a synthetic promoter containing three binding sites for the Eyeless/PAX6 transcription factor and was generously provided by Ernst Wimmer [14]; the construct drives high-level expression of eGFP specifically in retinal tissue and is abbreviated here as *eyeless-GFP*. Certain strains were obtained from the Bloomington Drosophila Stock Center (BDSC), including strain y[1] w[*]; P{w[+mC] = elav-Switch.O}GSG301 (BDSC#43642), which is abbreviated here as *y; Elav-GS*, strain y[1] w[*]; P{tubP-GAL4}LL7/TM3 (BDSC#5138), abbreviated here as *tub-GAL4*, strain y[1] w[*]; wg[Sp-1]/CyO; *P{Mhc-Switch.O}GSG314-2* (BDSC#43641), abbreviated here as *Mhc-GS*, strain *w[1118]; P{UAS-MitoTimer}3* (BDSC#57323), abbreviated here as *UAS-MT* [15,16], and strain y[1] w[*]; *wg[Sp-1]/CyO*, *P{Wee-P.ph0}Bacc[Wee-P20]*; *P{y[+t7.7] w[+mC] = 20XUAS-6XmCherry-HA}attP2* (BDSC#52268), abbreviated here as *UAS-mCherry*. The *w[1118]* strain (BDSC#5905) is the isogenized version (*w[1118]-iso*; *2-iso*; *3-iso*) that was previously cured of Wolbachia [10]. The *UAS-eGFP* strain employed is abbreviated as *Ultra-GFP* and contains multiple copies of a *UAS-2xeGFP* construct on both the second and third chromosomes and has been previously described [1]. To generate mated females, age-synchronized virgin females at 1–2 days of age were combined with young (3–14 day old) male *w[1118]* flies in regular food vials, with 20 females and 20 males per vial, for 48 h, after which the males were removed.

### 2.3. Generation of Multi-Copy Strains

Generation of Tet-ON strain REDA: The Tet-ON system driver construct rtTA consists of the *Actin5C* promoter driving expression of the reverse tetracycline trans-activator protein called rtTA [1]. The construct is inserted on the third chromosome, and the insertion strain is named *rtTA(3)E2*. The target construct contains a synthetic promoter consisting of seven Tet-operator (TetO) sequences fused to the core promoter sequences of the *hsp70* gene, driving expression of DsRED [17]. Two independent strains bearing insertions of this *TetO-DsRED* construct on the third chromosome were used, *TetO-DsRED[6]* and *TetO-DsRED[26B]* [17], each in the *w[1118]* genetic background. The target transgenic constructs bear the *mini-white+* marker gene and have light orange-colored eyes resulting from partial rescue of the *w[1118]* mutant phenotype. To increase the copy number of the target constructs, each strain was crossed to *P(ry+,Δ2-3)99B* transposase strain [18], and third chromosomes were isolated that had increased dosage of the *mini-white+* marker, as indicated by a more red eye color, and named *TetO-DsRED[6-7]* and *TetO-DsRED[26B-6]*, respectively. The increased copy number of the constructs was confirmed by crossing to the *rtTA(3)E2* strain, assaying progeny ± DOX treatment, and confirming a total two-fold increase in DsRED signal using fluorescence microscopy. The *TetO-DsRED[6-7]* and *TetO-DsRED[26B-6]* insertions were then recombined onto the same third chromosome, and the increased copy number was confirmed by again crossing to the *rtTA(3)E2* strain, assaying progeny ± DOX treatment, and confirming a total four-fold increase in DsRED signal using fluorescence microscopy. Finally, the chromosome bearing the *TetO-DsRED[6-7]* and *TetO-DsRED[26B-6]* insertions was recombined with *rtTA(3)E2* to generate a chromosome bearing all three insertions on the same third chromosome, balanced over *TM3 Sb* balancer, and in a y[*] w[*] genetic background; the resulting strain y[*] w[*]; *TetO-DsRED[6-7]*, *TetO-DsRED[26B-6], rtTA(3)E2/TM3 Sb*, which is abbreviated as “REDA”. The yellow mutant body color allows for increased detection of fluorescence from internal tissues relative to a wild-type body color.

Multi-copy mitoGFP strain: The strains *P{UAS-mito-HA-GFP.AP}2* and *P{UAS-mito-HA-GFP.AP}3* [19] were obtained from BDSC (BDSC#8442; BDSC#8443). Each strain was crossed to *P(ry+,Δ2-3)99B* transposase strain, and chromosomes were isolated that had increased dosage of the *mini-white+* marker strain, as indicated by a more red eye color, and named *UAS-mitoGFP[AP2-4]* and *UAS-mitoGFP[AP3-7]*, respectively. The increased copy number of the constructs was confirmed by crossing to the *tub-GAL4* strain and assaying progeny to confirm a two-fold increase in GFP signal using fluorescence microscopy. *UAS-mitoGFP[AP2-4]* and *UAS-mitoGFP[AP3-7]* were then combined into the same strain using double-balancer crosses to yield strain y[*] w[*]; UAS-mitoGFP[AP2-4]; UAS-mitoGFP[AP3-7]/TM3 Ser.

Multi-copy MitoTimer strain: The *UAS-MT* strain was crossed to the *P(ry+,Δ2-3)99B* transposase strain, and a third chromosome was isolated that had an increased dosage of the *mini-white+* marker, as indicated by a more red eye color, and named *UAS-MT[1]*. The increased copy number of the construct was confirmed by crossing to the *tub-GAL4* strain and assaying progeny to confirm a two-fold increase in green and red fluorescence signals using fluorescence microscopy. The *UAS-MT[1]* was crossed to the *P(ry+,Δ2-3)99B* transposase strain, and the third chromosome was isolated that had an increased dosage of the *mini-white+* marker strain, as indicated by a more red eye color, and named *UAS-MT[1-1]*. The increased copy number of the construct was confirmed by crossing to the *tub-GAL4* strain and assaying progeny to confirm a three-fold increase in green and red fluorescence signals using fluorescence microscopy (Figure S5).

### 2.4. Cameras and Filters

The 3D tracking and quantification of GFP fluorescence were carried out essentially as previously described [9]. Briefly, two video cameras (Grasshopper type GRAS-20S4C, Point Grey Research, Scottsdale, AZ, USA) were directed at the glass vial containing the fly. The vial was illuminated by two LED lights, and video was recorded at 30 fps. For GFP fluorescence tracking, blue LEDs were used as follows: The MF469-35 filters (Thorlabs, Inc., Newton, NJ, USA) were fixed in front of the blue LEDs to limit the light to a range of approximately 452 nm to 486 nm, which overlaps the eGFP absorption peak at approximately 488 nm. The MF525-39 filters were placed in front of the camera lenses to limit the light to the range of approximately 510 nm to 548 nm, which overlaps the emission peak for eGFP at approximately 509 nm. For DsRED tracking, green LEDs were used as follows: The TRITC excitation filters (MF542-20; Thorlabs, Inc.) were fixed in front of the green LEDs to limit the light to a range of approximately 532 nm to 552 nm, which overlaps the DsRED absorption peak at approximately 555 nm. The TRITC/CY3.5 emission filters (Thorlabs MF620-52) were placed in front of the camera lenses to limit the light to the range of approximately 595 nm to 645 nm, which overlaps the emission peak for DsRED at approximately 590 nm.

### 2.5. Vials, Media and Fly Handling for Video Assays

Assay of tissue-general eGFP in parallel in young and old, male and female flies. Flies were maintained as adults on NutriFly Grape Agar Premix media (Genesee Scientific 47-102), adjusted to 0.5% Casein protein (Sigma/Aldrich, St. Louis, MO, USA); this is to reduce background green fluorescence from the gut that can result from the standard *Drosophila* media. For assay, 6 flies were knocked into an empty 4 dram glass shell vial (Kimble 60965-4, Wayne, PA, USA), without anesthetization, on a foam mouse pad to avoid damage to vials. The bottom of the vial was covered with a round piece of black filter paper to reduce glare, and the top of the vial was covered with a glass coverslip.

Assay of eGFP, mitoGFP and DsRED in various tissue patterns. Clear glass shell vials (4 mL; ThermoFisher, Waltham, MA, USA) were filled with 500 µL media. The media was NutriFly Grape Agar Premix (Genesee Scientific 47-102, San Diego, CA, USA), adjusted to 0.5% Casein protein (Sigma/Aldrich) and adjusted to 0.25% wgt/vol blue dye #1. This media was used to reduce background fluorescence. The media was adjusted to a final concentration of 200 µg/mL mifepristone for the Gene-Switch system or to 640 µg/mL doxycycline for the Tet-ON system. To introduce flies, the flies were lightly anesthetized with CO_2_ gas, knocked into the vial, and the vial covered at the top with a small piece of clear tape (Grey Parrot brand crystal clear office tape) to prevent flies from escaping without compromising visibility. Flies were passaged to fresh vials every other day (using light CO_2_ anesthesia). When not being assayed, all flies were kept in an incubator at 25 °C, with the exception of mitoGFP flies, which were maintained at 22 (±0.5) °C.

### 2.6. In Vivo Bortezomib Treatment

Virgin female progeny of *Actin-GS-255B* x *ultraGFP* cross were placed in vials with media adjusted to a final concentration of 20 µM bortezomib. The control group media received an equal volume of ethanol vehicle. All flies were also exposed to 200 µg/mL mifepristone in the media for 48 h starting from the first day of videos. Videos were taken every day for 11 days. Flies were switched to fresh ± drug vials every 48 h through the end of the experiment.

### 2.7. In Vivo Cycloheximide Treatment

Virgin female progeny of the *Actin-GS-255B* x *ultraGFP* cross were treated with cycloheximide added to food vials as an aqueous solution. Flies were treated with cycloheximide beginning 7 days before the start of video recordings, at an initial age of 1–3 days. Flies in the low-concentration group were maintained on media adjusted to a final concentration of 5 µM cycloheximide, and the high-concentration group was maintained on media adjusted to a final concentration of 10 µM cycloheximide. Flies in the control group were maintained on vials adjusted with an equal volume of water vehicle. Beginning with the initial video recording, all flies were transferred to vials adjusted to 200 µg/mL mifepristone, in addition to any cycloheximide. Videos were taken every day for 7 days. After 48 h, flies were removed from mifepristone and transferred to new ± cycloheximide vials; flies continued to be switched to fresh drug vials every 48 h through the end of the experiment.

### 2.8. Microscope Image Capture Assay and Statistical Analysis

Flies with eGFP expression were maintained on NutriFly Grape Agar Premix (Genesee Scientific 47-102), adjusted to 0.5% Casein protein (Sigma/Aldrich). Flies with red fluorescent protein expression were maintained in regular fly media vials. Flies were transferred to fresh vials every other day without anesthetization. For image capture, three flies at a time were anesthetized with CO_2_ on a fly pad (Genesee Scientific), positioned on their side, and fluorescence images were generated using the Leica MZFLIII fluorescence microscope and Spot imaging system. Fly fluorescence was quantified using Image J version 1.54 software [20] and selecting the desired fly body regions as ROI using the free-hand drawing tool. For each time point, >10 flies per sample were quantified, and the data average ± standard deviation was calculated. The ln(fluorescence) versus time was plotted and analyzed using Prism 9 to conduct linear regression and generate half-life values.

### 2.9. In Vitro Proteasome Assay

In vitro degradation of protein by the proteasome was assayed essentially as previously described [10]. Young (3–4 day old) virgin female flies from the eyeless-GFP strain were used to generate extracts containing abundant eGFP. Whole flies were homogenized using a motorized pestle in an extraction buffer composed of 50 mM Tris, 25 mM KCl, 10 mM NaCl, 1 mM MgCl_2_, and 1 mM DTT at pH 7.5. Three groups of 20 flies each were homogenized in 500 µL of buffer. After homogenization, each sample was subjected to 3 rounds of a freeze/thaw cycle consisting of 5 min in dry ice followed by 5 min in a room-temperature water bath. After 3 cycles the samples were centrifuged for 2 min at 10,000× *g* in a 4 °C cold room. The supernatants from the 3 replicates were pooled and then diluted 1:4 in the buffer solution. Next, 180 µL aliquots of the diluted extract were added to black 96-well plates (Greiner Bio-One 82050-728, Kremsmünster, Austria), and 20 µL of either pure ethanol (control) or 250 µM Bortezomib (experimental group) was added to each sample. Purified recombinant eGFP (Cell Biolabs STA-201, San Diego, CA, USA) at 1 mg/mL in PBS was diluted 1:5, 1:10 and 1:15 with PBS, and 200 µL of each sample was loaded into the plate in duplicate as controls. This plate was then introduced to the plate reader pre-warmed to 37 °C. GFP fluorescence was quantified using the BioTek Synergy H4 Hybrid Multi-Mode Microplate Reader (Agilent, Santa Clara, CA, USA) in the USC Dornsife NanoBiophysics Core facility (Los Angeles, CA, USA). Readings were taken at 30 s intervals at 395 nm excitation/510 nm emission to detect GFP fluorescence.

### 2.10. Generation of Partially Purified eGFP Extract

Young virgin female flies of the *eyeless-GFP* strain were anesthetized with CO_2_ on the fly pad, and the heads were removed using a razor blade. A total of 80 heads were combined with 1 mL PBS, homogenized and subjected to 3 rounds of freeze/thaw, centrifuged as described above, and the supernatant used as extract. The extract was diluted 1:2, 1:4, 1:8, and 1:16 in PBS and added to the plate at 180 µL per well. Three replicates of each concentration were supplemented with 20 µL of 250 µM bortezomib, and the controls were supplemented with 20 µL of ethanol vehicle. Reads were conducted using a plate reader as described above, at room temperature (23.5 ± 0.5 °C). GFP fluorescence was quantified using a BioTek Synergy H4 Hybrid Multi-Mode Microplate Reader. For all experiments, readings were taken at 30 s intervals at 395 nm excitation/510 nm emission to detect GFP fluorescence.

### 2.11. Video Analysis Software and Statistical Analysis

Videos were generated and analyzed essentially as previously described [9]. The software SaveImageToAviEx_v142.exe is used to record videos at 30 frames per second. The desired number of frames to capture is input by the user, and the resulting video from each camera is recorded and saved as .avi video files. The FluoreScore suite is next used to process these videos into quantitative data. The software FluoreScoreGUI is used to analyze representative videos, select a fluorescence threshold for fly detection, select the ROI, and create a mask if needed to omit any areas with background fluorescence from the analysis. Next, the software template.bat file is used and can be edited for each experiment to indicate the chosen threshold for fluorescence detection, the coordinates for the ROI, and which mask files to utilize, if any. The template.bat file is the only file the user edits and runs directly. It calls the software FluoreScoreCMDV2.exe, which analyzes the videos and derives fluorescence values for the flies in each frame of video, and calls the software SqueezeData_V1.1.exe, which combines the data from the two cameras and filters out signal below the input threshold. The final output is .csv files with total fly fluorescence for each frame of video. The software Cymito_v5.R uses the data from the .csv files to create plots of average fluorescence versus time for each group in the experiment. This software also generates a summary .csv file, with the data in an easy-to-read format, including columns for the average daily fluorescence for every vial, average across all vials, and standard deviation. The data are then plotted as average ln(fluorescence) versus time using Prism 9 to conduct linear regression and generate half-life values. Linear models were also constructed using values from the replicate vials, rather than a single average for each time point, but demonstrated no advantage in identifying differences between groups or constructing lines with a good fit. To conduct ANOVA, the half-life values along with information about the age and sex of the flies were organized into a table, which was then read into R. The R lm function was used to construct a linear model relating half-life to age and sex (including interaction), and then the ANOVA function was used to run the statistical test. Both functions are available in base R version 4.4.

## 3. Results

### 3.1. In Vivo Assay of eGFP Half-Life Using the Gene-Switch System

**Figure 1:**
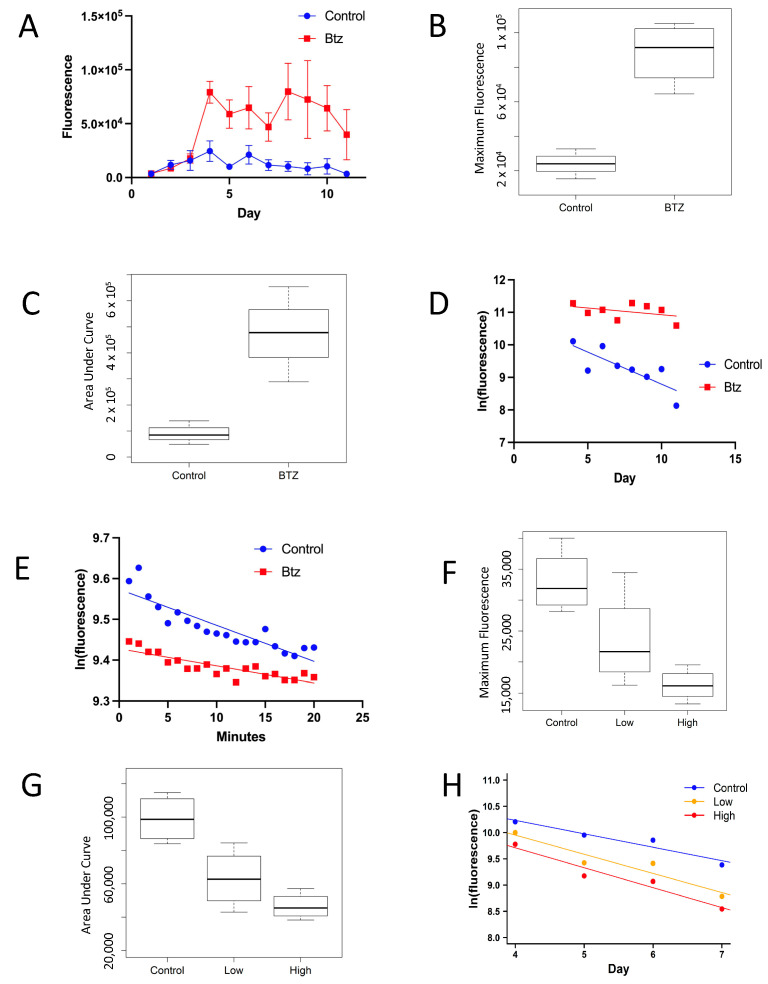
Effect of proteasome inhibitor bortezomib and translation inhibitor cycloheximide. eGFP was expressed in free-moving flies using the Gene-Switch system. The driver transgene is *Actin-GS-255B*, in which the tissue-general *Actin5C* gene promoter drives the expression of the Gene-Switch transcription factor. The target construct is *UAS-eGFP*, where the UAS promoter sequences bind Gene-Switch to drive the expression of eGFP. (**A**) Fluorescence assay of pulsed eGFP expression in free-moving virgin females (control; blue symbols) and in virgin females treated with 20 µM bortezomib (Btz; red symbols). ID#070722. (**B**) The peak expression level for control and bortezomib-treated flies at day 4, from (**A**). *p* = 0.00315. (**C**) Area under the curve (AUC) quantification of eGFP fluorescence for control and bortezomib-treated flies in (**A**). *p* = 0.00239. (**D**) Fluorescence decay from the peak for control and bortezomib-treated flies in (**A**). The control half-life is 3.5 days, and the Btz half-life is 17 days. *p* = 0.0489. (**E**) In vitro proteasomal degradation assay. Fluorescence decay of eGFP in fly extract (control; blue symbols) and in the presence of 20 µM bortezomib (Btz; red symbols). Control half-life 78 min, Btz half-life 165 min. *p* = 0.0004. ID#072922. Statistical tests are linear regression and ANCOVA; a statistical summary is presented in Table S1. (**F**) Fluorescence assay of pulsed eGFP expression in free-moving virgin females and in females pre-treated with low concentration (5 µM) or high concentration (10 µM) cycloheximide. The peak levels of eGFP fluorescence for control and cycloheximide-treated flies are presented (Experiment ID #081323). Peak levels in the high drug concentration group are significantly lower than the control group (*p* = 0.003284). The low-concentration group did not significantly differ from the control (*p* = 0.09583) or high drug concentration (*p* = 0.1586). (**G**) Area under the curve (AUC) quantification of eGFP fluorescence for control and cycloheximide-treated flies (Experiment ID #081323). Control AUC was significantly different from low drug concentration (5 µM; *p* = 0.02091) and high drug concentration (10 µM; *p* = 0.001728). (**H**) Fluorescence decay of eGFP in control and cycloheximide-treated flies in (Experiment ID #081323). The slope for the control group was −0.2558 (half-life 2.71 days), the slope for low concentration was −0.3648 (half-life 1.90 days), and the slope for high concentration was −0.3801 (half-life 1.82 days). Slopes did not significantly differ (*p* = 0.4281776). Slopes compared with ANCOVA.

eGFP was expressed in free-moving flies using the Gene-Switch system [3]. The driver transgene was Actin-GS-255B, in which the tissue-general Actin5C gene promoter drives expression of the Gene-Switch transcription factor [1]. The target was multiple copies of the UAS-eGFP transgene (the “Ultra-GFP” strain), in which the UAS promoter sequences bind Gene-Switch to drive expression of eGFP [1]. When flies containing this driver and target are fed the trigger drug mifepristone, this activates the Gene-Switch transcription factor to produce high-level, tissue-general induction of eGFP [1]. eGFP expression in the flies was quantified using two video cameras (Figure S1), and analysis of the time course for eGFP induction shows that the level of eGFP expression peaks approximately 24 h after the end of the 48 h period of drug treatment (Figure 1A and Figure S8D). To determine the in vivo eGFP half-life, the period from the peak of expression to the subsequent minimum was transformed to the natural log and analyzed by linear regression (Figure 1D). This yielded an in vivo half-life for eGFP in this experiment of 3.5 days.

### 3.2. Bortezomib Increases eGFP Half-Life In Vivo and In Vitro

To confirm that the loss of eGFP fluorescence with time is due to protein degradation, the dependence of fluorescence decay on normal proteasome function was assayed. To determine if cytoplasmic eGFP is degraded by the proteasome, the proteasome inhibitor drug bortezomib was employed [21]. Bortezomib was fed to flies at 20 µM in the media, coincident with the induction of eGFP expression by mifepristone. Notably, eGFP expression in flies treated with bortezomib reached maximum levels approximately three times greater than controls, consistent with inhibited degradation due to bortezomib (Figure 1A,B). Quantification of area under the curve (AUC), a measure of total eGFP accumulation, confirmed a 5.6-fold greater eGFP expression in the bortezomib-treated group relative to the control group across the entire assay period (Figure 1C). Consistent with these results, plotting ln(fluorescence) versus time reveals a greater in vivo half-life for eGFP in the bortezomib-treated flies (17 days) relative to controls (3.5 days; Figure 1D; Supplementary Table S1). Increased area under the curve for eGFP accumulation in the presence of bortezomib was confirmed in two additional experiments (Supplementary Figure S2).

To further confirm degradation of eGFP by the proteasome and inhibition by bortezomib, an in vitro proteasome assay was used (Figure 1E) [10]. Extracts prepared from flies expressing eGFP were incubated in the absence and presence of 20 µM bortezomib, and the change over time in eGFP fluorescence was measured using a plate reader. The rate of decay of fluorescence in fly extracts is more rapid than that in living animals, and therefore half-life was measured in minutes rather than days. Plotting ln(fluorescence) versus time again revealed inhibition of eGFP degradation by bortezomib, as indicated by increased eGFP half-life, consistent with proteasome-dependent degradation (Figure 1E; Supplementary Table S1). We note that the absolute eGFP fluorescence detected by plate reader assay was reduced in the presence of bortezomib; however, the magnitude of the inhibitory effect was roughly equal across different sources and concentrations of eGFP, including dilutions of partially purified eGFP (Supplementary Figure S3A,B), as well as purified recombinant eGFP and the starting extract used for proteasome assay (Supplementary Figure S3C), indicating that half-life calculations are not significantly affected (see Section 4).

### 3.3. Cycloheximide Decreases eGFP Expression In Vivo

To determine if the in vivo assay could also capture changes in protein synthesis, the translation inhibitor cycloheximide was fed to flies at concentrations of 5 µM and 10 µM in the media. A pulse of eGFP expression was then induced with mifepristone. The peak level of eGFP fluorescence achieved was significantly lower in the drug-treated groups compared to controls (Figure 1F). Consistent with this, quantification of AUC revealed reduced accumulation of eGFP in the cycloheximide-treated groups across the entire assay period (Figure 1G). The half-life of eGFP in the presence and absence of cycloheximide treatment was calculated from the slope of ln(fluorescence) versus time. The eGFP half-life did not differ significantly between the groups, indicating that the decreased eGFP accumulation observed was driven by decreased synthesis (Figure 1H). These results support the conclusion that the in vivo assay provides a relatively accurate measure of in vivo protein half-life.

### 3.4. Half-Life Values of Different Fluorescent Proteins

Having validated the assay, several different fluorescent proteins were chosen to illustrate this assay’s ability to measure the decay rates of proteins whose stabilities might vary and to calculate both longer and shorter half-life values. The fluorescent proteins used in these experiments were eGFP, mitoGFP, DsRED, mCherry, and MitoTimer. Half-life values were calculated under different conditions, including age, sex, and mating status of the flies, as well as targeting different tissues or subcellular compartments.

**Figure 2:**
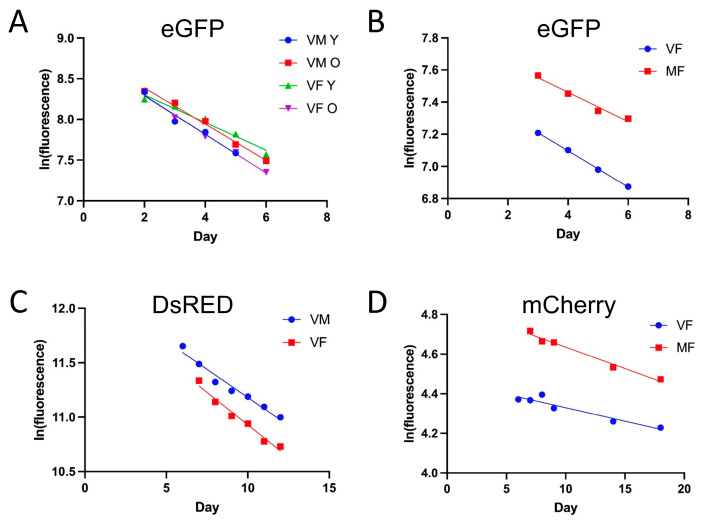
Half-lives of different fluorescent proteins. (**A**,**B**) Tissue-general expression of eGFP. The driver transgene is *Actin-GS-255B*, in which the tissue-general *Actin5C* gene promoter drives expression of the Gene-Switch transcription factor. The target construct is *UAS-eGFP*, where the UAS promoter sequences bind Gene-Switch to drive expression of eGFP. (**A**) Fluorescence decay of eGFP in young (Y; 6 days old) and old (O; 36 days old) virgin males (VM) and virgin females (VF). Half-life values in days: 2.9 (VM Y), 3.1 (VM O), 4.1 (VF Y), 2.9 (VF O). Experiment ID #092619. (**B**) Fluorescence decay in virgin females (VF) and mated females (MF). ID #121720. Half-life values in days: 6.2 (VF) and 7.6 (MF). Statistical summary for eGFP experiments presented in Supplementary Table S1. (**C**) Fluorescence decay of DsRED in free-moving flies, using the Tet-ON system. The driver transgene is *rtTA(3)E2*, in which the tissue-general *Actin5C* promoter drives expression of the rtTA transcription factor. The target transgene is *TetO-DsRED*, where the TetO promoter sequences drive expression of DsRED. Virgin males (VM) and virgin females (VF). *p* = 0.2552. ID #111920. Half-life in days: 6.7 (VM), 5.8 (VF). (**D**) Fluorescence decay of mCherry in flies anesthetized during microscope image capture using the Gene-Switch system. The driver transgene is *Actin-GS-255B*, and the target transgene is *UAS-mCherry*. Virgin females (VF) and mated females (MF). *p* = 0.0319. ID#072221. Half-life in days: 51 (VF), 32 (MF). Statistical summary for mCherry presented in Supplementary Table S5. Statistical tests are linear regression and ANCOVA.

**Figure 3:**
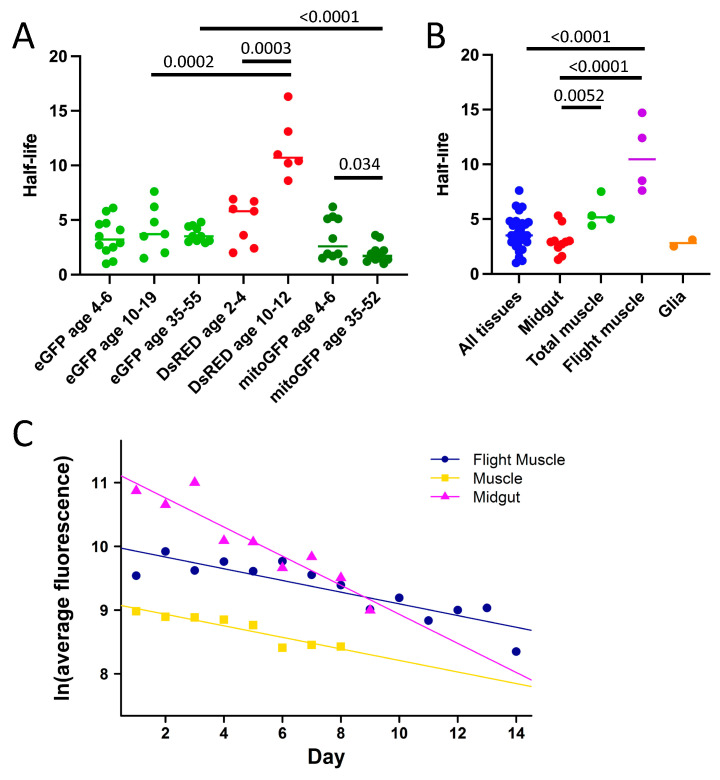
Half-life differences across age and tissue types. (**A**,**B**) Data were analyzed across all video and microscope assays. The statistical test is an unpaired, two-tailed *t*-test, and *p*-values for comparisons are presented above the plots. (**A**) Half-life of fluorescent proteins in flies of different age groups. The fluorescent proteins and the age range of the flies are indicated below the *X*-axis. In each case fluorescent protein expression is tissue-general. Both eGFP and mitoGFP were expressed using the *Actin-GS-255B* driver and the Gene-Switch system, whereas DsRED was expressed using the *rtTA(3)E2G* driver and Tet-ON system. (**B**) Half-life of eGFP expressed in various tissues using the Gene-Switch system. “All tissues” indicates tissue-general expression using the *Actin-GS-255B* driver. Midgut is *5966-GS* driver, total muscle is *Mhc-GS* driver, flight muscle is *88F-GS* driver, and glia is *REPO-GS* driver. (**C**) Examples of fluorescence decay of eGFP targeted to muscle and midgut of mated females. In each case the target construct is *UAS-eGFP*. eGFP fluorescence decay is plotted for flight muscle (using driver *88F-GS*, Experiment ID #100221), total muscle (using driver *Mhc-GS*, Experiment ID #092021), and midgut (using driver *5966-GS*, Experiment ID #111021). The slope of eGFP decay in the midgut (half-life 3.0 days) differed from flight muscle (half-life 7.6 days, *p* = 0.003159) and muscle (half-life 7.5 days, *p* = 0.0006211), whereas no significant difference was detected between flight muscle and total muscle (*p* = 0.9671537). Statistical tests are linear regression and ANCOVA.

The first experiments were designed to compare cytoplasmic eGFP degradation rates in males and females, at both younger and older ages, with tissue-general expression. Five independent experiments were conducted comparing young and old virgin males and young and old virgin females, with all four groups assayed in parallel. The young fly starting ages were 5–6 days, and the old fly starting ages were 35–55 days. The average eGFP half-life was 3.9 days in young males, 3.9 days in young females, 4 days in old males, and 3.4 days in old females. No statistically significant relationship between eGFP half-life and age was detected when analyzing the entire dataset using ANOVA (Figure 2A; Supplementary Table S6). It is notable that eGFP half-life in young males and females appears particularly variable (Figure 3A; Supplementary Table S1 and Figure S4A), and this may be one reason a significant effect of age was not detected when analyzing the entire dataset. However, when ANOVA analysis was limited to only the flies >30 days of age, a significant increase with age was detected (*p* = 0.039) (Supplementary Figure S4B). No significant relationship was detected between eGFP half-life and either sex or female mating status (Figure 2B, Supplementary Table S1).

A COX8 mitochondrial targeting sequence fused to the N-terminus of eGFP results in mitoGFP [19]. *UAS-mitoGFP* construct insertions were mobilized to increase copy number and combined into the same strain (Section 2). When crossed to the tissue-general *Actin-GS-255B* driver, this yielded sufficient mitoGFP signal to allow video assay of half-life, giving an average half-life of 2.6 days (Supplementary Figure S6 and Table S3). Interestingly, mitoGFP half-life appeared slightly reduced in old flies relative to young flies (Figure 3A), and mitoGFP in old flies had a shorter half-life than did cytoplasmic eGFP (Figure 3A). No consistent effects of sex or mating status on mitoGFP half-life were observed (Supplementary Table S3).

The half-life of the red fluorescent protein DsRED was assayed using the Tet-ON conditional gene expression system, in which the *actin5C* gene promoter drives tissue-general expression of the doxycycline-activated transcription factor rtTA, targeting the construct *TetO-DsRED* in the presence of the drug (Supplementary Figure S9A,B). Different conditional expression systems were used for eGFP and DsRED in part due to a lack of reagents for Tet-ON. The time course for DsRED induction using Tet-ON revealed a peak of expression approximately 4–5 days after the 48 h period of drug treatment (Figure S8C,E). The average half-life of DsRED in video assays of young virgin males and virgin females was 7.9 days (Figure 2C; Supplementary Table S4). Notably, when analyzed across all video and microscope assays, the half-life of DsRED increased dramatically with age (Figure 3A), and the half-life of DsRED was significantly greater than that of eGFP in flies of comparable age (Figure 3A). No consistently significant difference in DsRED half-life was observed between virgin males and virgin females or between virgin females and mated females (Supplementary Table S4).

Another red fluorescent protein, mCherry, was found to have a particularly long half-life. The Gene-Switch system and the tissue-general *Actin-GS-255B* driver were used to drive expression of mCherry, using a high copy number *UAS-mCherry* strain. Microscope assay yielded a half-life of 51 days in virgin females and 32 days in mated females (Figure 2D; Supplementary Table S5). Induction of mCherry expression using the GeneSwitch system was also observed using the glia-specific *REPO-GS* driver (Supplementary Figure S9C,D), as well as with other drivers. However, the video assays were complicated by high background and poor fit to exponential decay models, and half the assays did not detect any decay in red fluorescence (Supplementary Table S5).

Finally, another red fluorescent protein, the mitochondria-targeted MitoTimer, was also assayed [15,22]. A multi-copy *UAS-MitoTimer* strain was constructed (Supplementary Figure S5), and MitoTimer was expressed in neurons using the *Elav-GS* driver. Robust induction of red fluorescence was observed in video assays in both virgin males and virgin females, yielding a half-life of 4.8–6.7 days. There was no significant difference in half-life between the sexes (Supplementary Table S5).

### 3.5. Tissue-Specific Expression of eGFP

Protein half-life can vary by tissue [23,24]. To investigate possible differences in tissue-specific eGFP turnover rates, eGFP was expressed using several tissue-specific drivers, and half-life was assayed in virgin males, virgin females and mated females (Supplementary Table S2). When analyzed across all assays, the half-life of eGFP expressed in total muscle or flight muscle was significantly greater than that in midgut, and the half-life of eGFP in flight muscle was significantly greater than that observed with tissue-general expression (Figure 3B). Specifically, tissue-general expression of eGFP using the *Actin-GS-255B* driver yielded an average eGFP half-life of 3.7 days; targeting eGFP expression to the midgut using the *5966-GS* driver yielded an average half-life of 3.0 days; targeting eGFP to total muscle using the *Mhc-GS* driver yielded an average half-life of 5.6 days; targeting eGFP to flight muscle using the *88F-GS* driver yielded an average half-life of 10.8 days; and finally, targeting eGFP to glia using the *REPO-GS* driver yielded an average half-life of 2.8 days (Figure 3B). No consistent difference in eGFP half-life between virgin and mated females was observed with any driver (Supplementary Table S2).

To enable direct comparison of eGFP decay rates between tissues, data were normalized with regard to time, using the datasets with the largest number of points in the linear range. Consistent with the data presented above, the eGFP half-life in the midgut was smaller than that in total muscle or flight muscle in both mated females (Figure 3C) and virgin females (Supplementary Figure S7).

## 4. Discussion

Here, methods for measuring the half-life of fluorescent proteins in living flies are presented, which enable measurements of protein turnover under different conditions and in distinct tissues and subcellular locations. This assay captures changes in both protein degradation and synthesis. The half-life of eGFP was increased by the proteasome inhibitor bortezomib, both in vivo and in vitro, indicating proteasomal degradation of eGFP. Consistent with the conclusion that eGFP is degraded by the proteasome, bortezomib has previously been shown to inhibit GFP degradation in cultured human H1299 carcinoma cells [25], and proteasome inhibitors MG132 and Z-L3-VS inhibited GFP degradation in cultured HeLa cells [26]. It was observed that in the plate reader assay, absolute eGFP fluorescence was slightly lower in the presence of bortezomib. The absolute magnitude of this effect was approximately equal across different concentrations of eGFP, with a larger percent change at lower concentrations (Supplementary Figure S3). This indicates that the observed effect, a decrease in half-life in the presence of bortezomib, was unlikely to be an artifact of this inhibition, because a preferential decrease in eGFP fluorescence at lower concentrations would tend to produce a steeper rate of decay, and the effect observed with bortezomib is in the opposite direction. Thus, the presence of this inhibitory effect should not affect our conclusions. The inhibitory effect was not detected in the video experiments. The accumulation of eGFP in vivo was decreased by the protein synthesis inhibitor cycloheximide, without affecting half-life.

DsRED protein was expressed in a tissue-general pattern using the Tet-ON system and *Actin5C* gene promoter and had a generally greater half-life than was observed for tissue-general expression of eGFP using the Gene-Switch system and *Actin5C* gene promoter. For example, the average half-life of eGFP across the video assay experiments was 4.1 days, whereas the average half-life of DsRED across the video assay experiments was 7.8 days (Supplementary Tables S1, S2 and S4). Consistent with these observations, a previous study of cultured *Drosophila* S2 cells reported a half-life for eGFP of approximately 3.8 days and a half-life for DsRED of approximately 8 days [27]. The similarity between the half-life values previously obtained using cultured *Drosophila S2* cells and the half-life values obtained here for in vivo *Drosophila* assays supports the accuracy of our method for measuring in vivo fluorescent protein half-life, as well as its ability to discern differences in the decay rate between different proteins. Limited degradation of mCherry was detected, and examples of decay rates for eGFP, DsRED and mCherry in young virgin females are plotted from peak to minimum on the same *X*-axis scale for comparison (Supplementary Figure S10).

Differences in the decay rates for fluorescent proteins can be exploited for various uses in research. For example, the Repressible Dual Differential Marker (ReDDM) method for lineage tracing takes advantage of the longer half-life of H2B-RFP (red fluorescent protein) relative to mCD8-GFP (green fluorescent protein) to distinguish stem cells from their differentiated progeny [28]. The half-life of DsRED increased rapidly between eclosion and day 12 of age, with statistically significant increases detected for each of virgin females, mated females, and virgin males. In the future, it may be of interest to ask if the half-life of DsRED continues to increase at greater ages.

Increased transgene copy number resulted in proportionally increased levels of fluorescent protein expression, as expected (for example, see Figure S5). This increased fluorescent protein expression is not expected to affect fluorescent protein stability. This is supported by several experiments in which similar half-life values were calculated for the same fluorescent protein despite differences in the maximum fluorescence value (for example, see Figure 2B). There was no indication of any saturation of the video camera assays or fluorescence microscope assays, such as fluorescence curves that are truncated at the top (for examples, see Supplemental Figure S8), indicating that the assays are within the linear range. Whereas differences in light penetration might yield somewhat lower fluorescence intensity from internal tissues, we were able to reproducibly assay fluorescent protein half-life in internal tissues such as the gut and nervous system (see Tables S2 and S5). In the future, increasing the input light intensity might further increase the ability to assay subsets of internal cells.

Half-life also varied by tissue, with greater eGFP half-life observed in muscle relative to other tissues. Cytoplasmic eGFP had a similar half-life in young flies when expressed in a tissue-general pattern and when targeted to midgut enterocytes and to glial cells (summarized in Figure 3B). In contrast, eGFP half-life was longer in young flies when targeted to muscle tissue (Figure 3B). The relatively longer half-life of eGFP in *Drosophila* muscle tissue may be related to observations that muscle tissue shows preferential accumulation of protein aggregates and induction of hsp70 protein relative to other adult tissues during aging [29,30]. These results appear similar to the mouse, where muscle tissue was reported to exhibit generally slower protein turnover than other tissues such as liver [23]. Limited effects were observed for *Drosophila* sex and female mating status. Taken together, the data indicate the in vivo assays are promising tools for the study of protein degradation regulated by protein sequence, subcellular compartment, tissue and small molecules.

Limitations of this study include some asymmetry of the experiments. For example, eGFP has been assayed only using the Gene-Switch system, and DsRED has been assayed only using the Tet-ON system. One reason for this is the relative lack of reagents for the Tet-ON system. Successful expression of eGFP with Tet-ON has been reported in the past, but the tetO-GFP strains are no longer extant [17]. For both the Gene-Switch and Tet-ON systems, the protein half-life is calculated from the peak of expression to the subsequent minimum. Because the peak in protein expression occurs >24 h after the cessation of drug treatment, it seems unlikely that the differences in the measured half-lives of eGFP and DsRED are a consequence of using different systems but rather are due to inherent differences in the half-lives of the two proteins. In the future, it may be of interest to generate additional fluorescent protein target constructs for the Tet-ON system to allow for direct comparison of half-life values between different proteins without the possible confounding effects of different transgenic systems.

A limitation of the Gene-Switch system is that the mifepristone used to trigger fluorescent protein expression can have significant physiological effects. In mated females, mifepristone reduces progeny production and midgut size and greatly increases median life span [31]. Limiting mifepristone treatment to 48 h is expected to limit confounding effects. However, it is possible the effects of even brief mifepristone treatment are one reason that differences in eGFP half-life between virgin and mated females were not detected using Gene-Switch. Other conditional gene expression systems each have their own limitations. For example, using heat shock gene promoters and a heat pulse is problematic due to the effects of heat on *Drosophila*. In particular, increased temperature reduces the physiological changes associated with mating in female flies, including eliminating the effect of mating on lifespan and midgut size, making heat shock induction unsuitable for comparisons of virgin and mated females [31]. Moreover, increased temperature shortens lifespan in *Drosophila* and can affect protein turnover, altering both synthesis and degradation [32,33]. The DOX (doxycycline) drug used to trigger the Tet-ON system has been found to have small effects on mitochondrial translation and gene expression [34]. Similarly, the Q-system for conditional gene expression is confounded by the fact that the triggering drug, quinic acid, has pleiotropic effects on aging and life span [35,36]. Therefore, the use of any conditional gene expression system requires careful consideration of the possible side effects of the system activator.

Additional limitations of the video assay include some variability in results, particularly for the youngest flies. Reasons for this likely include some unequal lighting throughout the vial and some unequal coverage of all regions by the video cameras, combined with fly movement, which is greater in young flies. In future experiments, this variability might be reduced by adding a third camera to improve vial coverage, adding additional lighting, and/or by lengthening video recording time to lessen the impact of fly movement.

## Data Availability

The datasets supporting the conclusions of this article are included within the article/Supplementary Materials and its additional files. Software used for video assays is available upon publication at https://github.com/johntower/Tracking-Paper-2024 (accessed on 27 May 2025).

## Supplementary Materials

The following supporting information can be downloaded at https://www.mdpi.com/article/10.3390/insects16060583/s1, Figure S1: Setup of video assay; Figure S2: Effect of bortezomib in vitro, replicate assays; Figure S3: Effect of bortezomib on fluorescence plate reader assay; Figure S4: Summary of half-life values for young and old virgin males and virgin females assayed in parallel; Figure S5: Increased copy number of transgenes produced by P element transposition; Figure S6: Degradation rate of tissue-general mitoGFP; Figure S7: Fluorescence decay of eGFP targeted to muscle and midgut of virgin females; Figure S8: Examples of time course for fluorescent protein expression; Figure S9: Examples of fluorescent protein expression in flies cultured in the absence and presence of drug; Figure S10: Comparison plot for half-life of eGFP, DsRED and mCherry in young virgin female flies; Table S1: Tissue-general eGFP expression in young and old virgin males and virgin females; Table S2: Tissue-general and tissue-specific expression of eGFP; Table S3: Tissue-general expression of mitoGFP; Table S4: Tissue-general expression of DsRED using the Tet-ON system; Table S5: Analysis of mCherry and MitoTimer; Table S6: ANOVA summary.

## Abbreviations

eGFP	enhanced green fluorescent protein
DsRED	*Discosoma* species red fluorescent protein
LED	Light-emitting diode
COX8	cytochrome c oxidase subunit 8
UAS	upstream activating sequence
GS	Gene-Switch
rtTA	reverse tetracycline trans-activator
tetO	Tet-operator
