# Supplementary material for: Tissue-Specific Fluorescent Protein Turnover in Free-Moving Flies
Source: Insects. 2025 May 31;16(6):583. doi: 10.3390/insects16060583 (PMC12193387; doi:10.3390/insects16060583)
Supplement: Supplementary file 1 [file insects-16-00583-s001.zip › insects-3559289-supplementary.pdf]

Supplementary materials

Tissue-specific fluorescent protein turnover in free-moving flies

Katherine S. Bell, Sebastian Ko, Sam Ali, Brett Bognar, Michael Khmelkov, Nick Rau, Oscar Peng, Mavi Eyuboglu, John Paine, Andy Tong, Anuj Saria, Siddharth Agrawal, Kelvin J.A. Davies, John Tower

Supplementary Data Tables

**Experiment ID#**	**Age**	**Type**	**Slope**	**R sq.**	**$t^{1/2}$**	***p***
080719 Ultra/255B	4	VM	-0.2781	0.9846	2.5 days	0.0077
	38	VM	-0.1559	0.963	4.4 days	
	4	VF	-0.149	0.9248	4.7 days	
	38	VF	-0.1551	0.9762	4.5 days	
082119 Ultra/255B	5	VM	-0.1128	0.9703	6.1 days	< 0.0001
	35	VM	-0.1987	0.9979	3.5 days	
	5	VF	-0.198	0.9907	3.5 days	
	35	VF	-0.2317	0.9937	3.0 days	
111219 Ultra/255B	6	VM	-0.1198	0.9257	5.8 days	0.0168
	55	VM	-0.1634	0.9738	4.2 days	
	6	VF	-0.1513	0.991	4.6 days	
	55	VF	-0.2203	0.9902	3.1 days	
092619 Ultra/255B	6	VM	-0.239	0.9683	2.9 days	0.0414
(**Fig 2A**)	36	VM	-0.2226	0.9897	3.1 days	
	6	VF	-0.1701	0.9688	4.1 days	
	36	VF	-0.2363	0.9949	2.9 days	
090919 Ultra/255B	5	VM	-0.3136	0.9687	2.2 days	0.0014
	44	VM	-0.1432	0.9893	4.8 days	
	5	VF	-0.2589	0.9844	2.7 days	
	43	VF	-0.2005	0.9523	3.5 days	
070722 Ultra/255B	10	VF (-)Btz	-0.1971	0.6404	3.5 days	0.0489
(**Fig 1A-D**)	10	VF (+)Btz	-0.04159	0.1706	17 days	
072922 Plate reader	4	VF (-)Btz	-0.00883	0.7987	78 mins	0.0004
(**Fig 1E**)	4	VF (+)Btz	-0.00418	0.7366	165 mins	
081323 Ultra/255B (**Fig 1F-H**)	9	VF (-)Chx	-0.2558	0.9271	2.71 days	0.4282
	9	VF (+)Chx (5μM)	-0.3648	0.9036	1.90 days	
	9	VF (+)Chx (10μM)	-0.3801	0.9425	1.82 days	

Supplemental Table S1. Tissue-general eGFP expression in young and old virgin males and virgin females. Ultra = Ultra-GFP (multi-copy UAS-2xeGFP strain). 255B = Actin-GS-255B. VF = virgin female, MF = mated female, VM = virgin male. Btz = bortezomib, Chx = cycloheximide. Age given in days.

Experiment ID#	Tissue	Age	Type	Slope	R sq.	$t^{1/2}$	*p*
083021 Ultra/5966 microscope assay	Midgut	12	VF	-0.234	0.9991	3.0 days	0.0105
		12	MF	-0.1298	0.9377	5.3 days	
082921 Ultra/88F microscope assay	Flight muscle	12	VF	-0.0560	0.9425	12.4 days	0.5709
		12	MF	-0.047	0.8545	14.7 days	
120119 Ultra/255B	All	19	VM	-0.1884	0.966	3.7 days	0.6473
		19	VF	-0.1458	0.972	4.8 days	
121720 Ultra/255B	All	13	VF	-0.1121	0.9993	6.2 days	0.1286
(**Fig 2B**)		13	MF	-0.09095	0.9721	7.6 days	
091921 Ultra/255B	All	13	VF	-0.4488	0.8472	1.5 days	0.4645
		13	MF	-0.3486	0.707	2.0 days	
091721 Ultra/255B	All	5	VM	-0.5641	0.9906	1.2 days	0.1257
		5	VF	-0.7312	0.937	1.0 days	
090721 Ultra/5966	Midgut	17	VF	-0.2403	0.6383	2.9 days	0.9955
		17	MF	-0.2408	0.8045	2.9 days	
111021 Ultra/5966	Midgut	8	VF	-0.2605	0.7811	2.7 days	0.6038
		8	MF	-0.2282	0.8832	3.0 days	
090321 Ultra/5966	Midgut	8	VF	-0.5259	0.9754	1.3 days	0.2472
		8	MF	-0.4349	0.9892	1.6 days	
091121 Ultra/5966	Midgut	17	VF	-0.1448	0.4631	4.8 days	0.1364
		17	MF	-0.2927	0.759	2.4 days	
092021 Ultra/Mhc	Muscle	13	VF	-0.1373	0.7553	5.0 days	0.1048
		13	MF	-0.0918	0.7655	7.5 days	
090521 Ultra/Mhc	Muscle	17	VF	-0.1316	0.8458	5.3 days	0.4538
		17	MF	-0.1569	0.8476	4.4 days	
100221 Ultra/88F	Flight muscle	12	VF	-0.0817	0.877	8.5 days	0.6526
		12	MF	-0.0905	0.8657	7.6 days	
100621 Ultra/repo	Glia	10	VF	-0.2233	0.5732	3.1 days	0.5498
		10	MF	-0.2768	0.9014	2.5 days	

Supplemental Table S2. Tissue-general and tissue-specific expression of eGFP. Ultra = Ultra-GFP (multi-copy UAS-eGFP). 255B = Actin-GS-255B. 5966 = 5966-GS (Gene-Switch driven by midgut enterocyte-specific 5966-GS). 88F = Actin88F-GS (Gene-Switch driven by flight muscle-specific *Actin88F* promoter). Mhc = Mhc-GS (Gene-Switch driven by muscle-specific Mhc promoter). repo = repo-GS (Gene-Switch driven by glial-specific *repo* gene promoter). VF = virgin female, MF = mated female, VM = virgin male. Age given in days.

Experiment ID#	Age	Type	Slope	R sq.	$t^{1/2}$	*p*
4D1	4	VM	-0.3653	0.8824	1.9 days	0.3526
	4	VF	-0.3653	0.9423	1.9 days	
4D3 (**Fig S6A**)	4	VM	-0.1121	0.807	6.2 days	0.5246
	4	VF	-0.137	0.7676	5.1 days	
35D5 (**Fig S6B**)	35	VM	-0.4493	0.9733	1.5 days	0.5395
	35	VF	-0.5694	0.8183	1.2 days	
52D10 (**Fig S6C**)	52	VM	-0.4397	0.9254	1.6 days	0.5715
	52	VF	-0.4859	0.9906	1.4 days	
52D11	52	VM	-0.3145	0.6717	2.2 days	0.9982
	52	VF	-0.3142	0.7831	2.2 days	
52D13	52	VM	-0.2051	0.8758	3.4 days	0.792
	52	VF	-0.1937	0.8119	3.6 days	
52D14	52	VM	-0.3634	0.8887	1.9 days	0.8198
	52	VF	-0.3919	0.8532	1.8 days	
52D2	52	VM	-0.5039	0.8677	1.4 days	0.0818
	52	VF	-0.7142	0.936	1.0 days	
M4	6	VF	-0.4163	0.9115	1.7 days	0.016
	6	MF	-0.1367	0.8783	5.1 days	
M2 (**Fig S6D**)	6	VF	-0.4604	0.7391	1.5 days	0.5666
	6	MF	-0.5733	0.7553	1.2 days	
M3	6	VF	-0.2106	0.8406	3.3 days	0.3572
	6	MF	-0.1311	0.7405	5.3 days	

Supplemental Table S3. Tissue-general expression of mitoGFP. VF = virgin female, MF = mated female, VM = virgin male. Age given in days.

Experiment ID#	Age	Fly type	Slope	R sq.	$t^{1/2}$	*p*
080821 Microscope assay	12	VF	-0.02173	0.7532	32 days	0.1272
	12	MF	-0.04081	0.7679	17 days	
080921	12	VF	-0.081	0.707	8.6 days	0.7162
	12	MF	-0.06695	0.2307	10.4 days	
081021	12	VF	-0.06781	0.5343	10.2 days	0.8425
	12	MF	-0.06273	0.6238	11.0 days	
122020	2	VF	-0.1151	0.896	6.0 days	0.0619
	2	MF	-0.195	0.9839	3.6 days	
112920	3	VF	-0.3485	0.5914	2.0 days	0.7196
	3	MF	-0.2823	0.9016	2.4 days	
070721	10	VM	-0.04249	0.7636	16.3 days	0.6278
	10	VF	-0.05278	0.5937	13.1 days	
111920	2	VM	-0.1032	0.9664	6.7 days	0.2552
**(Fig 2C)**	2	VF	-0.1195	0.9723	5.8 days	
091621	4	VM	-0.1003	0.8449	6.9 days	0.3226
	34	VM	-0.05267	0.5723	13.1 days	

Supplemental Table S4. Tissue-general expression of DsRED using Tet-ON system. VF = virgin female, MF = mated female, VM = virgin male. Age given in days.

**Experiment ID#**	**Tissue**	**Age**	**Type**	**Slope**	**R sq.**	$t^{1/2}$	***p***
072221 mCherry/255B Microscope assay (**Fig 2D**)	All	5	VF	-0.01369	0.9023	51 days	0.0319
		5	MF	-0.0217	0.9802	32 days	
072421E mCherry/ElavGS	Neurons	6	VF	-0.00527	0.00186	131 days	0.6151
		6	MF	-0.03718	0.2568	19 days	
072421B mCherry/255B	All	6	VF	+0.04266	0.209	ND	0.355
		6	MF	-0.005947	0.00536	129 days	
072421R mCherry/REPO	Glia	6	VF	+0.01788	0.103	ND	0.84
		6	MF	+0.00954	0.0128	ND	
030123 MT/ElavGS	Neurons	3	VM	-0.10333	0.589	6.71 days	0.8026
		3	VF	-0.12052	0.9285	5.75 days	
033023 MT/ElavGS	Neurons	6	VM	-0.11737	0.6701	5.9 days	0.7042
		6	VF	-0.14484	0.6225	4.79 days	

Supplemental Table S5. Analysis of mCherry and MitoTimer. 255B = Actin-GS-255B. REPO = repo-GS. MT = MitoTimer. ElavGS = Gene-Switch driven by neuronal *Elav* promoter. VF = virgin female, MF = mated female, VM = virgin male. Age given in days.

**All flies:**					
	Df	Sum Sq	Mean Sq	F value	Pr(>F)
Age	1	0.280839	0.280839	0.265453	0.613443
Sex	1	0.728154	0.728154	0.688261	0.418963
Age:Sex	1	0.125643	0.125643	0.118759	0.734872
Residuals	16	16.9274	1.057963	NA	NA
**Flies >30 day of age:**					
	Df	Sum Sq	Mean Sq	F value	Pr(>F)
Age	1	2.119553	2.119553	6.914957	0.039079
Sex	1	0.532296	0.532296	1.736596	0.235649
Age:Sex	1	0.452913	0.452913	1.477609	0.269803
Residuals	6	1.839103	0.306517	NA	NA

Supplemental Table S6. ANOVA summary

Supplementary Figures

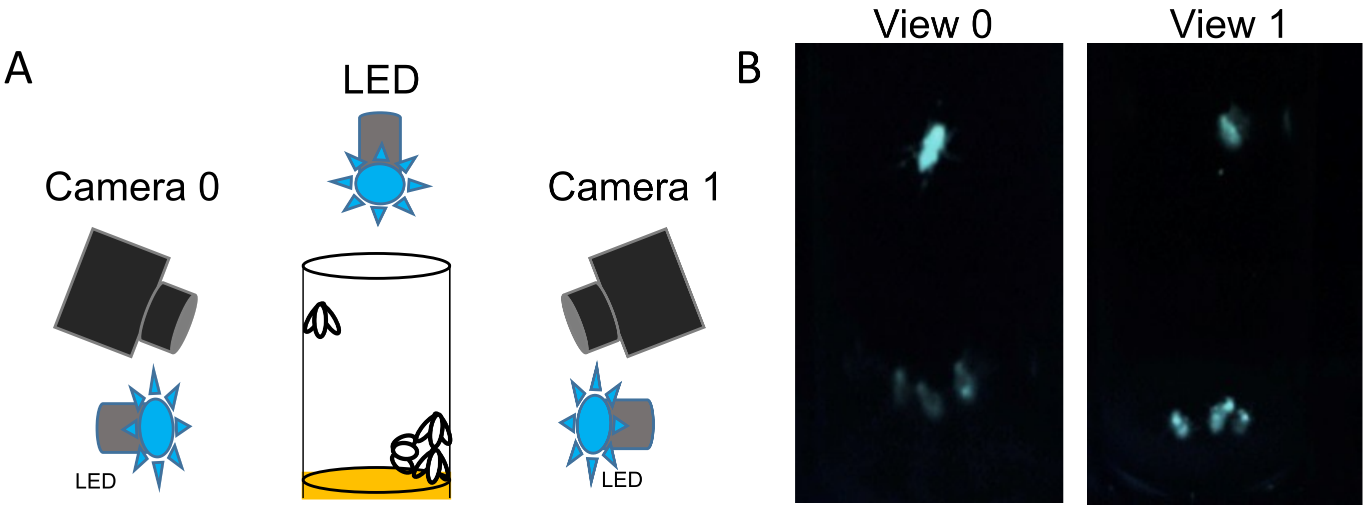

Figure S1. Setup of video assay. (A) Diagram of experimental set-up for video recording. Two video cameras (camera 0 and camera 1), and two LED lights are placed on opposite sides of a glass vial containing flies and media, and a third LED light is placed directly above the vial. **(B)** Example of a single video frame captured by camera 0 (view 0) and by camera 1 (view 1).

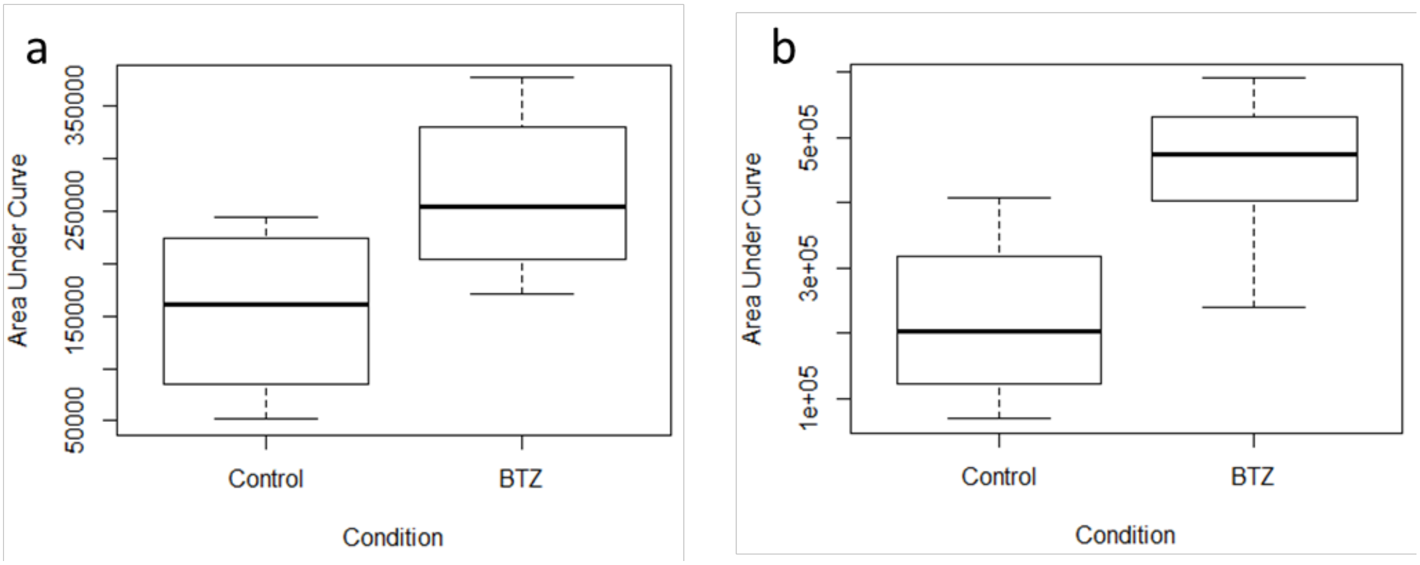

Figure S2. Effect of bortezomib in vitro, replicate assays. Fluorescence assay of pulsed eGFP expression in free-moving virgin females (Control; blue symbols), and in virgin females treated with 20µM bortezomib (Btz; red symbols). Area under the curve (AUC) quantification of eGFP fluorescence for control and bortezomib-treated flies, replicate experiments. **(a)** $p = 0.0435$. **(b)** $p = 0.0104$

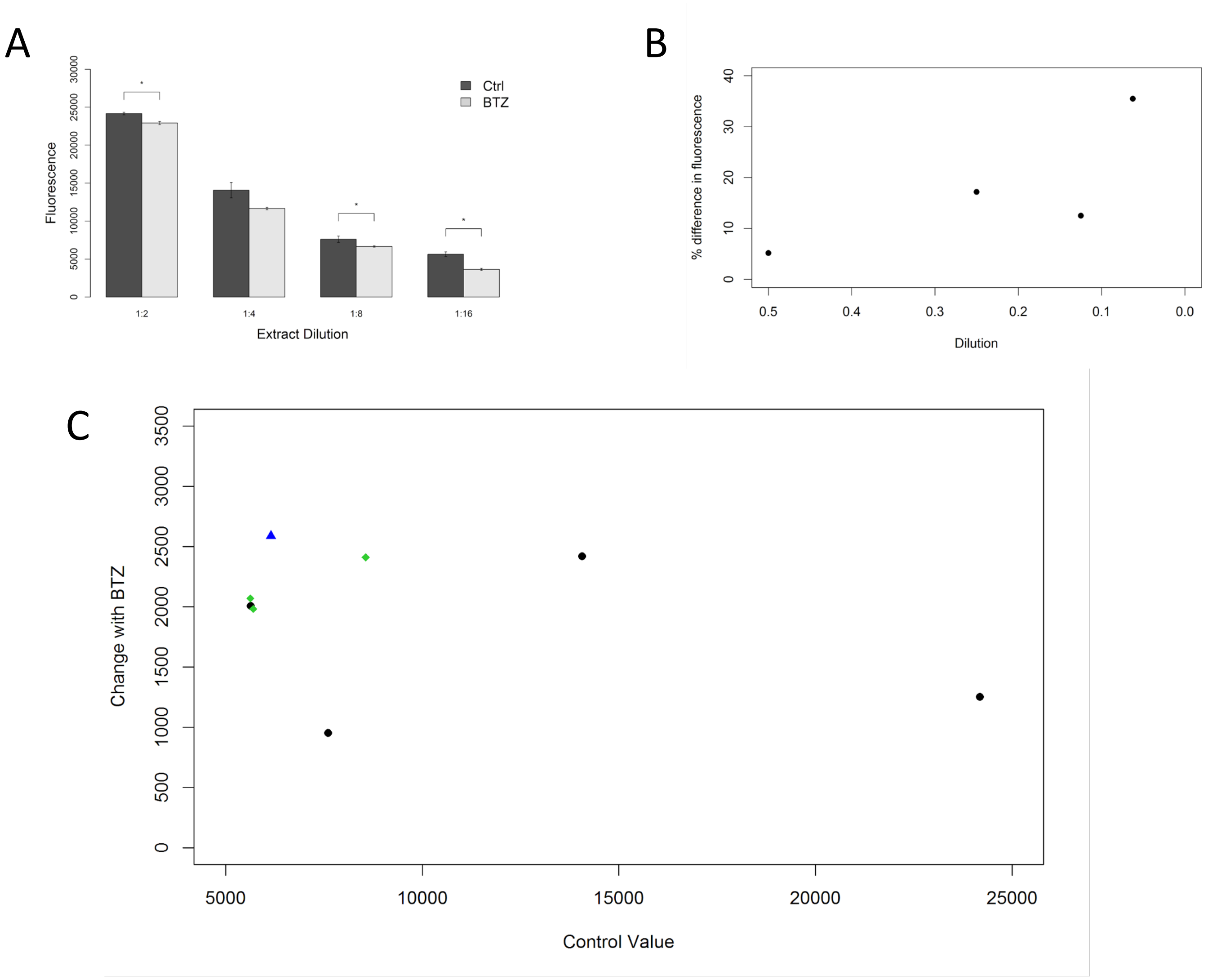

Figure S3. Effect of bortezomib on fluorescence plate reader assay
(A) Dilutions of partially-purified eGFP. Partially-purified eGFP extract was assayed at the indicated dilutions, in absence (Ctrl) and presence (BTZ) of 20µM bortezomib. Statistical test is unpaired, two-sided t test; asterisk indicates $p < 0.05$. **(B)** Percent change in fluorescence reading due to bortezomib for the samples shown in (a). **(C)** Absolute change in fluorescence reading due to bortezomib for various eGFP sources. The control fluorescence reading is plotted on the X axis, and the absolute decrease in fluorescence due to bortezomib is plotted on the Y axis. Black circles indicate dilutions of partially-purified eGFP, as shown in (A) and (B). Green diamonds indicate dilutions of purified recombinant eGFP. Blue symbol is whole-fly extract of flies expressing eGFP as used for proteasome assays.

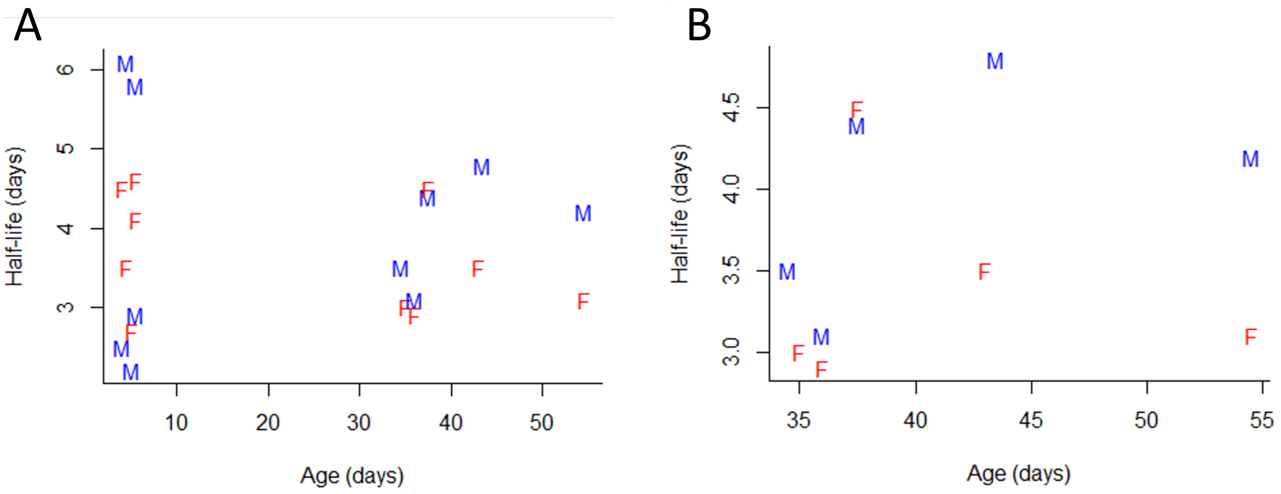

Figure S4. Summary of half-life values for young and old virgin males and virgin females assayed in parallel. Summary of eGFP half-life values for experiments listed in Table 1. M, males, F, females. **(A)** All flies. No significant relationship was found between half-life and age ($p = 0.6134$) or between half-life and sex ($p = 0.419$). **(B)** Flies >30 days of age. There is a significant relationship between half-life and age ($p = 0.03907$), and no significant relationship between half-life and sex ($p = 0.2356$). ANOVA summary presented in Supp Table S1.

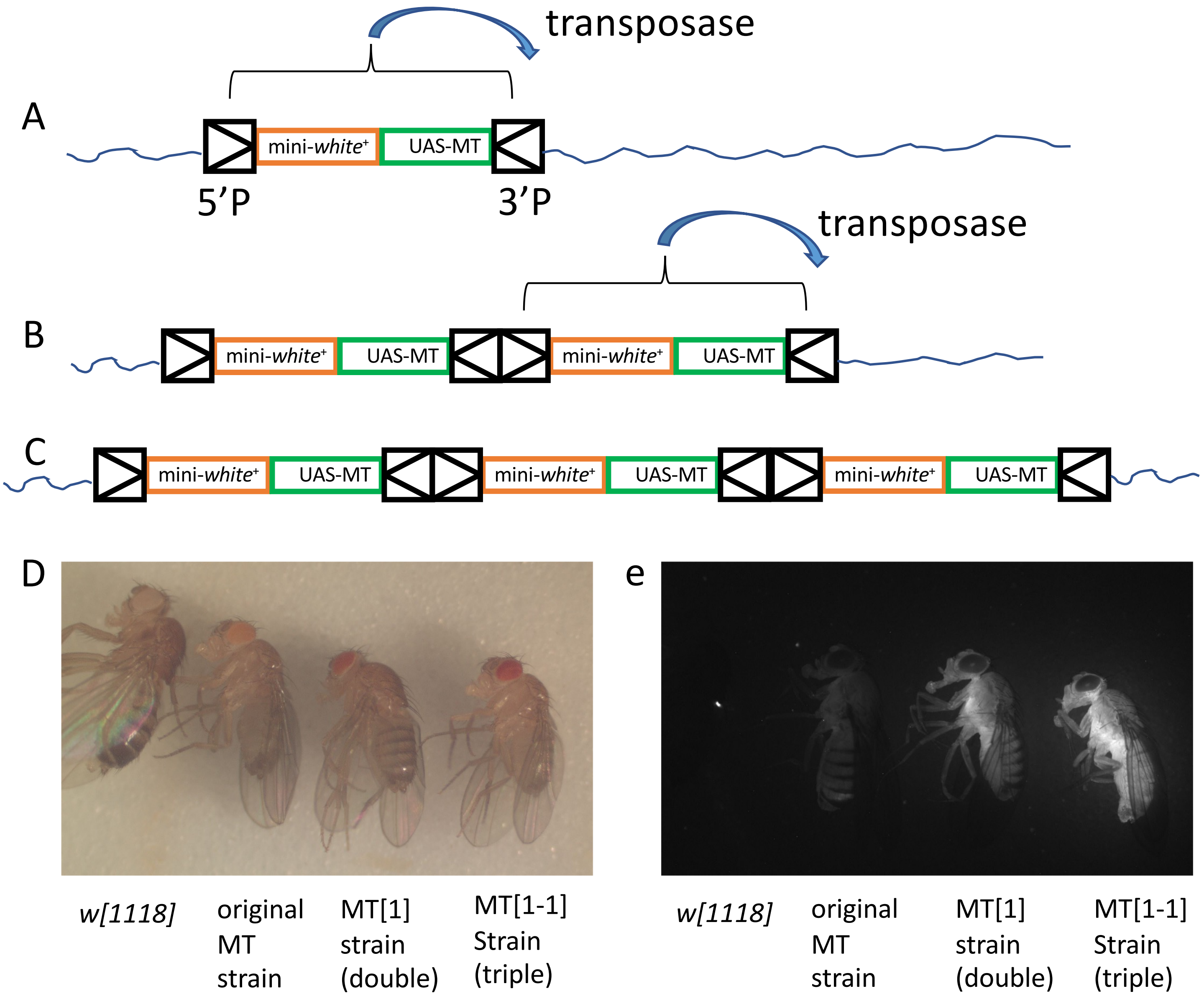

Figure S5. Increased copy number of transgenes produced by P element transposition. (A) Diagram of the MitoTimer transgenic insertion, indicating the 5' and 3' P element inverted repeats, the min-*white*+ marker gene sequences, and the UAS-MitoTimer (UAS-MT) gene sequences. **(B)** Crossing to a strain expressing P element transposase causes transposition of the construct, typically into the 5' or 3' P element end sequences, resulting in a duplication. **(C)** Crossing again to a strain expressing P element transposase causes a second round of transposition, resulting in 3 copies of the construct. **(D)** Increased expression of the mini-*white*+ marker indicated increased copy number. Left to right: visible light images are presented for the *w[1118]* control strain, the original MitoTimer strain, the MT[1] double-copy strain, and the MT[1-1] triple copy strain. The flies were generated by crossing each strain to the *w[1118]* strain to reveal the phenotypic series of mini-*white*+ eye phenotype in the progeny. **(E)** Increased expression of UAS-MitoTimer corresponding to increased copy number. Each strain was crossed to the Tubulin-GAL4 strain to cause constitutive expression of UAS-MitoTimer. Fluorescence signal in the red channel is presented in black and white image to reveal detail.

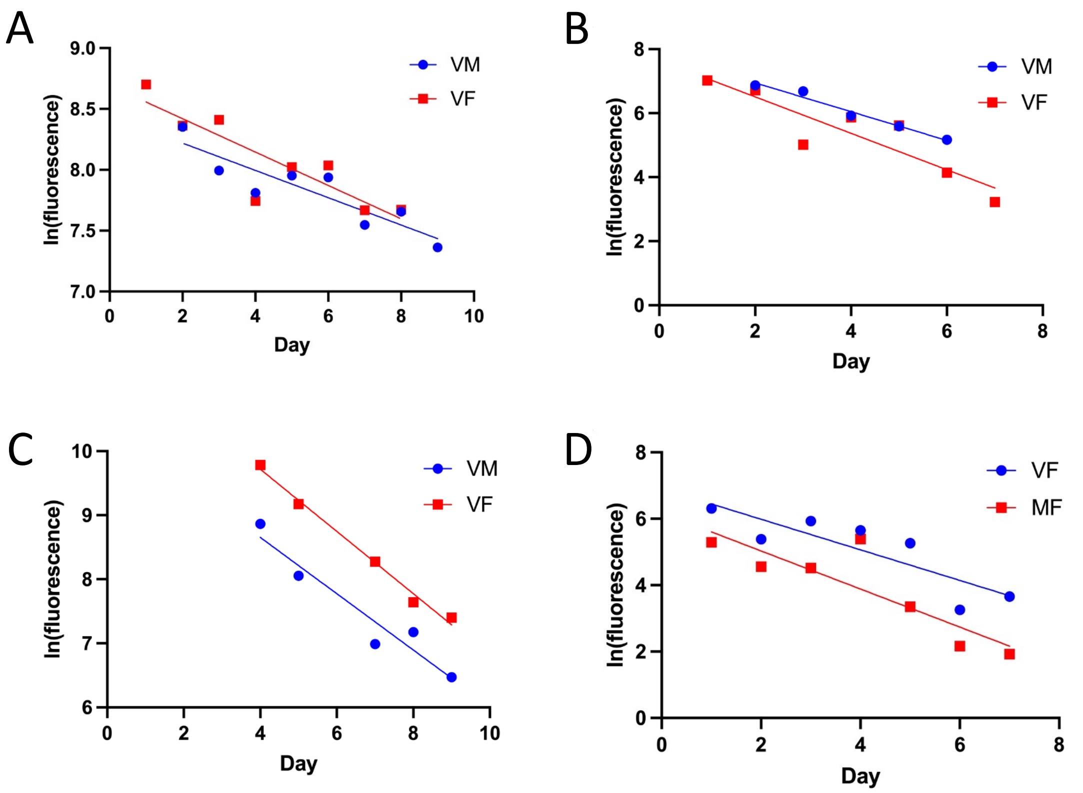

Figure S6. Degradation rate of tissue-general mitoGFP. Fluorescence decay of mitoGFP in free-moving flies. **(A)** Virgin males (VM) and virgin females (VF), 4 days old. $p = 0.5246$. ID#4D3. **(B)** Virgin males (VM) and virgin females (VF), 35 days old. $p = 0.5395$. ID#35D5. **(C)** Virgin males (VM) and virgin females (VF), 52 days old. $p = 0.5715$. ID#52D10. **(D)** Virgin females (VF) and mated females (MF), 6 days old. $p = 0.5666$. ID#M2. Statistical test is linear regression and ANCOVA; Statistical summary presented in Supplemental Table S3.

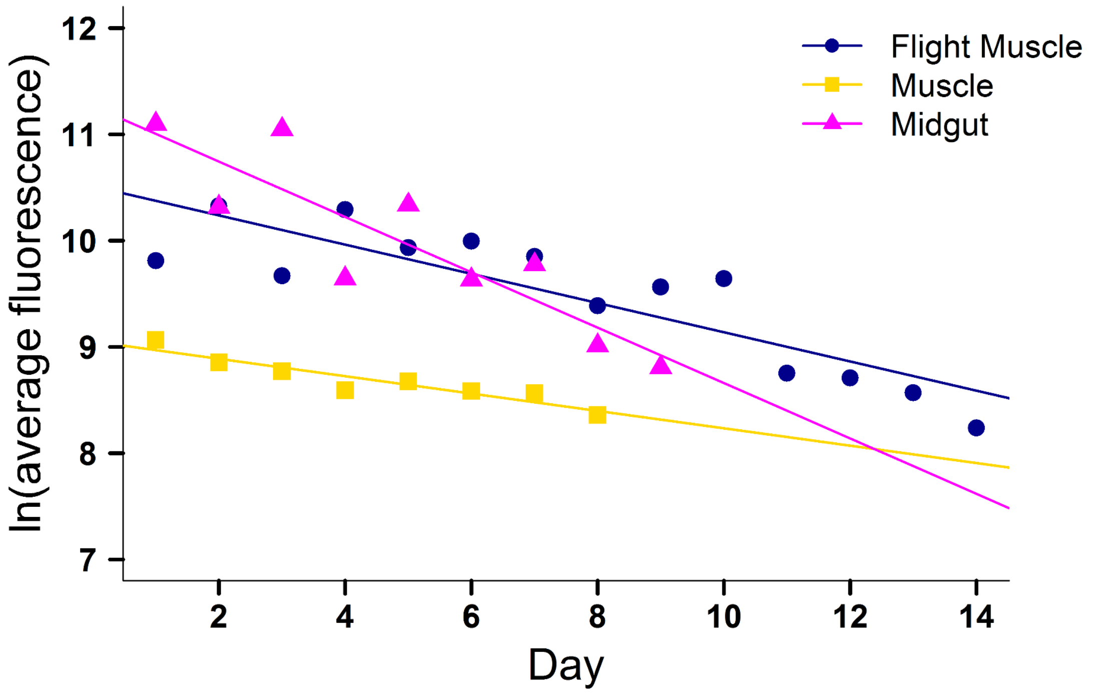

Figure S7. Fluorescence decay of eGFP targeted to muscle and midgut of virgin females. eGFP fluoresecence decay is plotted for flight muscle (88F driver, ID#100221), total muscle (Mhc-GS driver, ID#092021), and midgut (5966-GS driver, ID#111021). The slope of eGFP decay in midgut differed from flight muscle (p = 0.0113) and total muscle (p = 0.0311), whereas no significant difference was detected between flight muscle and total muscle (p = 0.256). Statistical test is linear regression and ANCOVA.

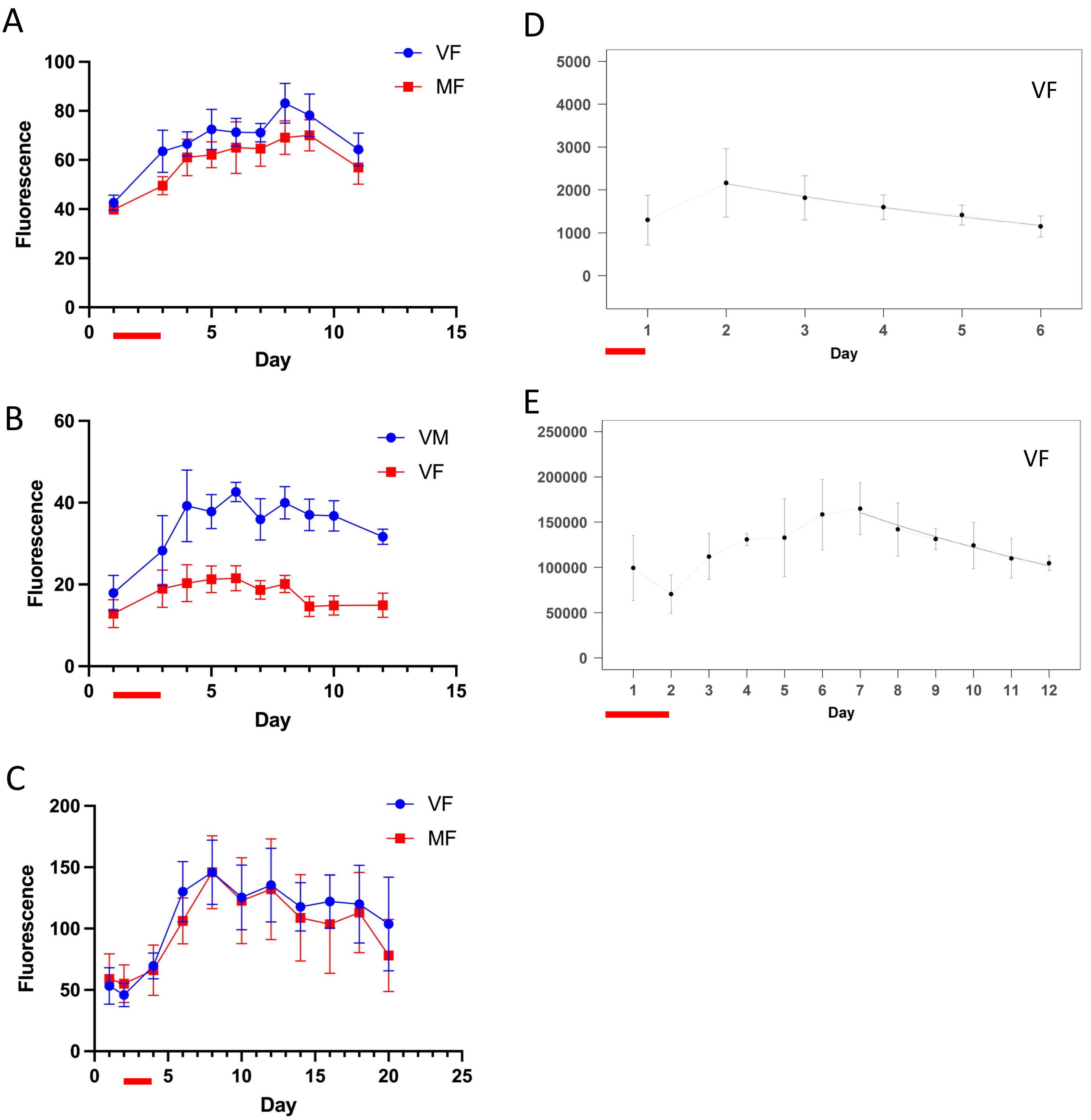

Figure S8. Examples of time course for fluorescent protein expression. Fluorescence intensity in live flies is plotted versus time in days. Red bars indicate the period of drug treatment. (**A-C**) Microscope assays. (**D-E**) Video assays. (**A**) eGFP targeted to flight muscle using *88F-GS* driver. (**B**) mitoGFP targeted to nervous system using *Elav-GS* driver. (**C**) DsRED expressed in tissue-general pattern using *actin5C-rtTA* driver and Tet-ON system. (**D**) eGFP expressed in tissue-general pattern using *Actin-GS-255B* driver and Gene-Switch system. Line indicates linear regression, half-life 4.6 days. (**E**) DsRED expressed in tissue-general pattern using *actin5C-rtTA* driver and Tet-ON system. Line indicates linear regression, half-life 7.6 days. VF, virgin female. VM, virgin male. MF, mated female.

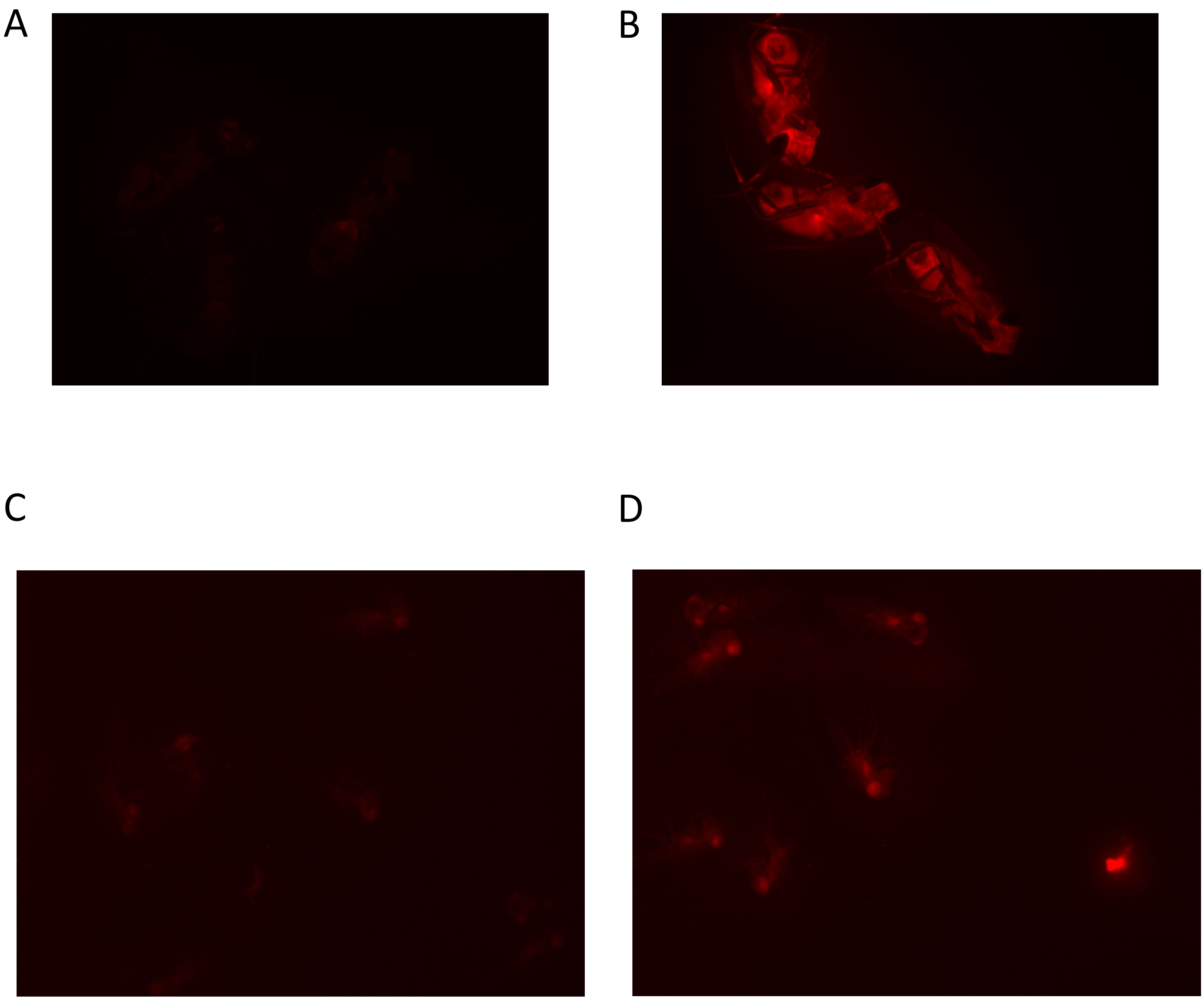

Figure S9. Examples of fluorescent protein expression in flies cultured in absence and presence of drug. (**A, B**) DsRED expressed in tissue-general pattern using the Tet-ON system with *actin5C-rtTA* driver and tetO-DsRED target, microscope assay. Three male flies are imaged in each group at 8 days after end of 48 hours drug treatment. (**A**) Minus drug group. (**B**) Plus drug group. (**C, D**) mCherry expressed in glia using the Gene-Switch system with driver *REPO-GS* and target *UAS-mCherry*. Seven mixed-sex flies are imaged in each group after 48 hours drug treatment. (**C**) Minus drug group. (**D**) Plus drug group.

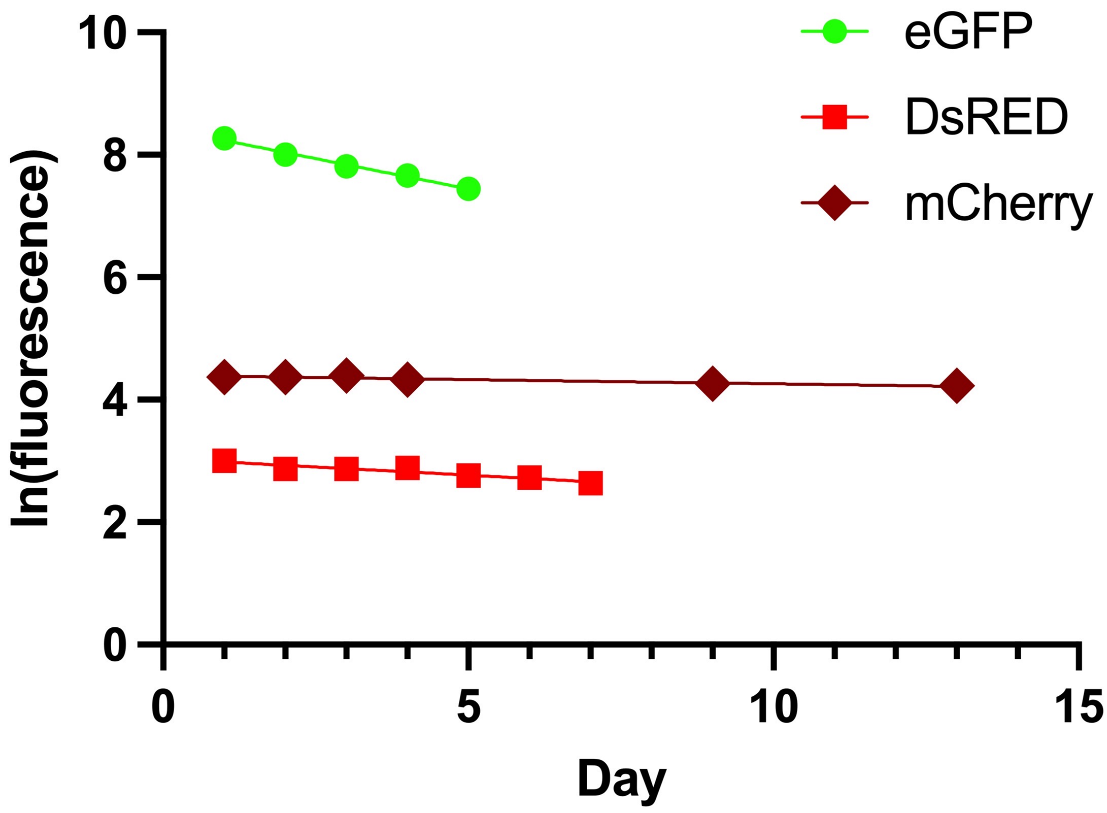

Figure S10. Comparison plot for half-life of eGFP, DsRED and mCherry in young virgin female flies. Ln(fluorescence) is plotted versus time in days, and lines indicate linear regression. Data is plotted from peak of expression to minimum in each case, with the peak set to day one of the plot. eGFP is experiment ID#082119, half-life is 3.5 days. DsRED is experiment ID#100120, half-life is 13 days. mCherry is experiment ID#072221, half-life is 51 days.
